# Supplementary material for: Patterned mechanical feedback establishes a global myosin gradient
Source: Nat Commun. 2022 Nov 17;13:7050. doi: 10.1038/s41467-022-34518-9 (PMC9672098; doi:10.1038/s41467-022-34518-9)
Supplement: Supplementary file 1 — Supplementary Information [file 41467_2022_34518_MOESM1_ESM.pdf]

Supplementary Information for

Patterned mechanical feedback establishes a global myosin gradient

Supplementary Figures

Figure S1: Image analysis pipeline
(A) Cross section of an embryo expressing sqhCherry approximately at the center of the A-P axis. White dots are surface detection. (B) Same cross section with central layer obtained from fitting the point cloud in A and shifting basally (red solid line). Red dashed lines represent the inner and outer most layers of the stack obtained by normally evolving the central layer. (C) Cross section of the image stack obtained in B. White dotted line represents the surface point cloud. (D) Red solid line represents the surface obtained by fitting the point cloud in (C). Red dashed lines represent the inner and outer most layers obtained by normally evolving the fitted surface. (E) Cartoon representation of the image stacks obtained from the cylinder and planar projection steps, respectively. Black dashed box indicates the information that will be included in the final maximum intensity projection image of 6 layers which is used for the analysis. (F) Pullback showing central germband region of surface shown in D 5 minutes before onset of VF. (G) cytoplasmic pool of F. (H) Junctional accumulation of F. (I) Quantitative comparison of myosin rate measurement as a function of DV position between an embryo expressing different fluorescent markers. N = 2. error bars are SD. (J) Example of a pullback displaying the full embryo surface as obtained from the procedure detailed above and shown in A-D. Each box shows the region displayed in the corresponding figure panel listed in the key below. Conventional pullback orientation is shown: anterior to the left, posterior to the right, ventral is top and bottom and dorsal is center.

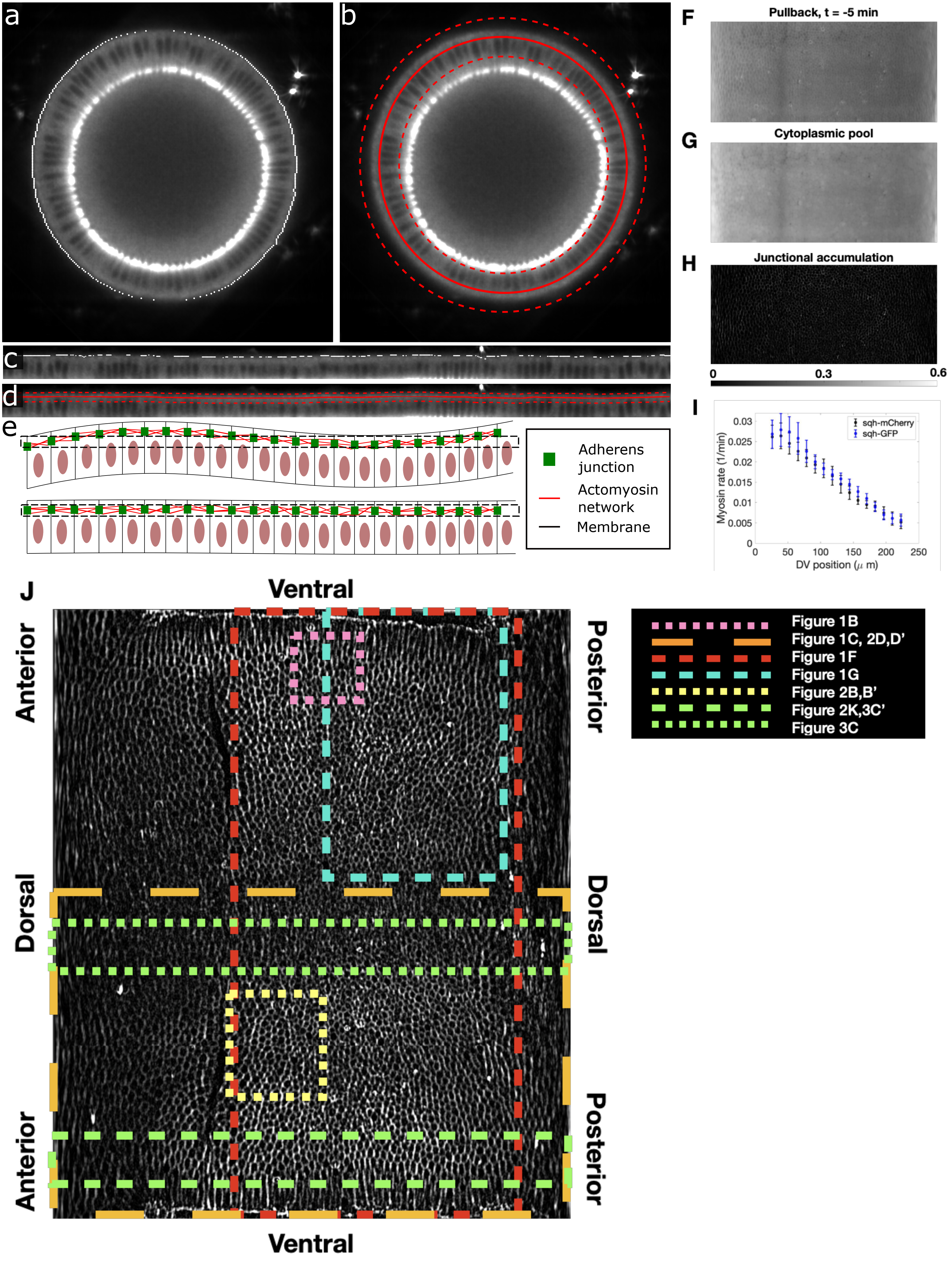

a
b
F
Pullback, t = -5 min
G
Cytoplasmic pool
H
Junctional accumulation
0
0.3
0.6
c
d
e
Adherens junction
Actomyosin network
Membrane
I
sqh-mCherry
sqh-GFP
Myosin rate (1/min)
DV position (μ m)
J
Ventral
Anterior
Posterior
Dorsal
Dorsal
Anterior
Posterior
Ventral
Figure 1B
Figure 1C, 2D,D'
Figure 1F
Figure 1G
Figure 2B,B'
Figure 2K,3C'
Figure 3C

Figure S2: Single junction tracking.
(A) Montage showing single edge tracking in the germband region of an embryo. Top is Ventral, left anterior. Each junction is labeled by a pseudo color. Individual frames are one minute apart. (B) Zoom on the dashed region in A. (C). As B, except using myosin marker 4µm below the adherens junction.

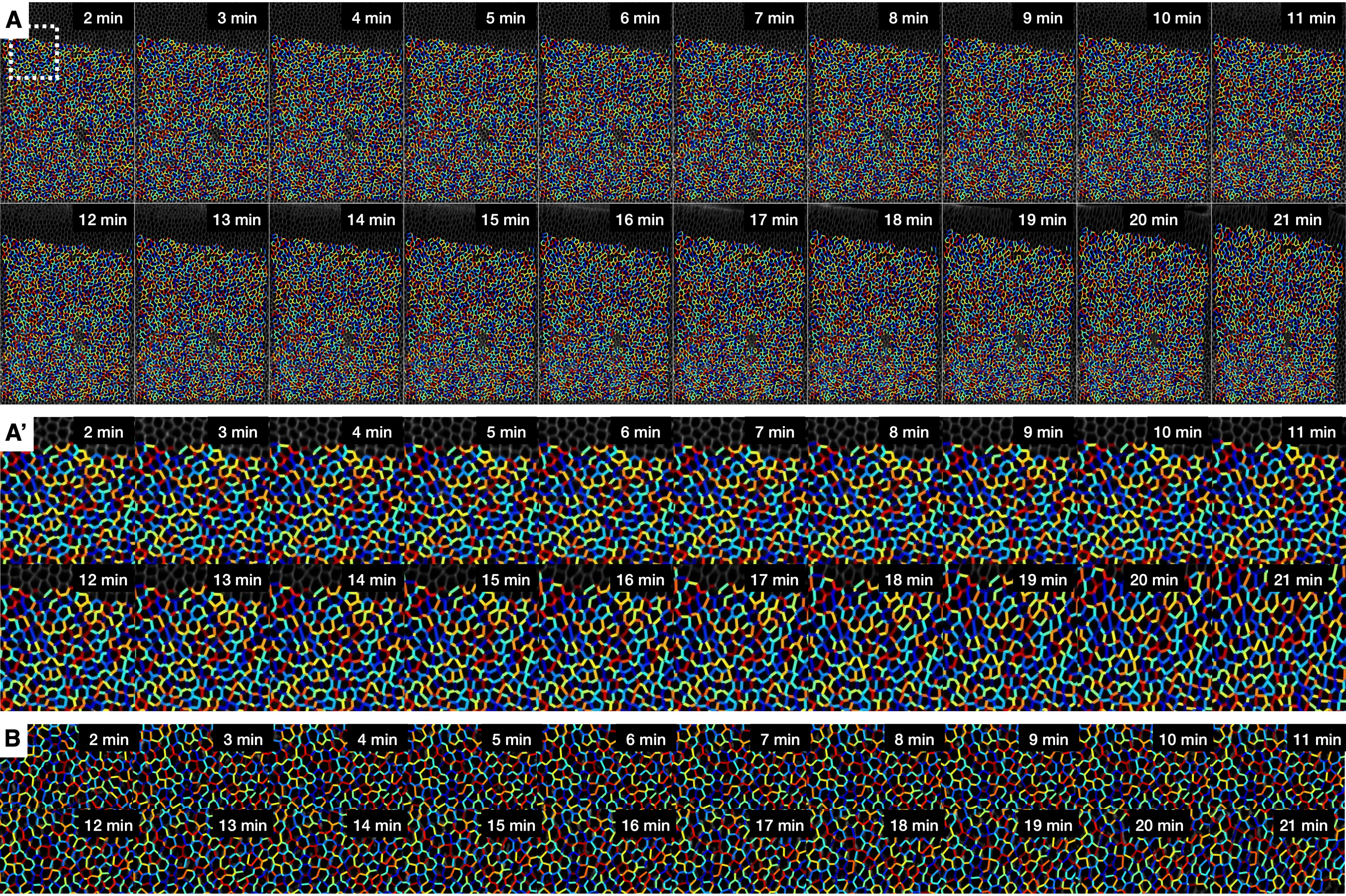
A
2 min
3 min
4 min
5 min
6 min
7 min
8 min
9 min
10 min
11 min
12 min
13 min
14 min
15 min
16 min
17 min
18 min
19 min
20 min
21 min
A’
B

Figure S3: Optogenetic activation scheme

(A) Whole embryo imaging with a 561 nm laser before activation. (B) Activation of the optogenetic construct by positioning the embryo in the sheet of a two-photon laser tuned to 940 nm. (C) Whole embryo imaging with a 561 nm laser following activation. (D,D') Example image of an activated embryo 30 seconds after activation viewed laterally D and dorsally D'. Red polygon indicates activation domain. (E) Whole embryo imaging with a 561 nm laser before activation. (F) Activation of the optogenetic construct by imaging only the head of the embryo while illuminating with a two-photon laser tuned to 940 nm. (G) Full embryo imaging with a 561 nm laser following activation. (H,H') Example image of an activated embryo 30 seconds after activation viewed laterally H and dorsally H'. Red box indicates optogenetic activation domain. (I) Schematic of timing of activation scheme with respect to landmark events of embryogenesis. The construct is transiently activated around the transition from cellularization to VF in regions outlined as above. (J) Normalized fluorescence intensity of activation signal shown in H. Red bar indicates posterior most position of the red box shown in H.

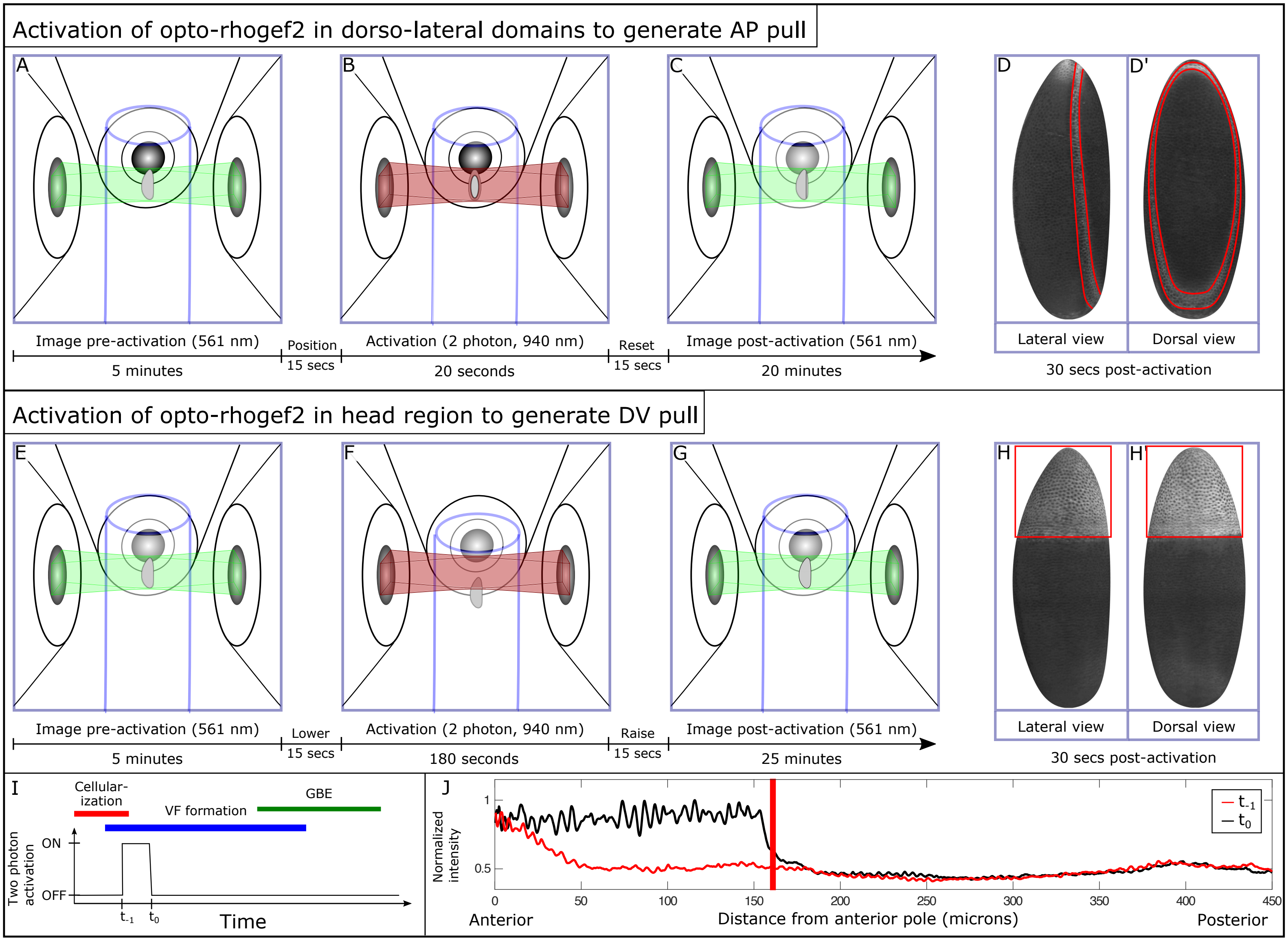

Activation of opto-rhogef2 in dorso-lateral domains to generate AP pull
A
B
C
D
D'
Image pre-activation (561 nm)
Position
15 secs
5 minutes
Activation (2 photon, 940 nm)
20 seconds
Reset
15 secs
Image post-activation (561 nm)
20 minutes
Lateral view
Dorsal view
30 secs post-activation
Activation of opto-rhogef2 in head region to generate DV pull
E
F
G
H
H'
Image pre-activation (561 nm)
Lower
15 secs
5 minutes
Activation (2 photon, 940 nm)
180 seconds
Raise
15 secs
Image post-activation (561 nm)
25 minutes
Lateral view
Dorsal view
30 secs post-activation
I
Cellular-ization
VF formation
GBE
Two photon activation
ON
OFF
t-1
t0
Time
J
Normalized intensity
1
0.5
0
50
100
150
200
250
300
350
400
450
t-1
t0
Anterior
Distance from anterior pole (microns)
Posterior

Figure S4: Quantitative characterization of strain parallel to DV axis.

(A-A'') Quantification of junctional myosin accumulation across the entire surface of an embryo activated in several planes separated by 5 um steps. t=0 is first frame after activation, corresponds to approx. 5 minutes after CF formation. (A) Junctional myosin 1 minute prior to activation. (A') Junctional myosin 0.5 minutes after activation. White boxes indicate the 2 regions analyzed for (B-F). Red dashed lines outline regions of activation. (A''). Junctional myosin 6 minutes after activation. (B) Trajectories of cells in region 2 tracked for 5 minutes before activation. (B') Trajectories for the same cells in B tracked for the 5 minutes following activation. (C,C') Cells in region 2 color coded by their eccentricity 1 minute before C and 5 minutes after C' activation. (D) Junctional accumulation of myosin over time for region 1. (E) Junctional accumulation of myosin over time for region 2. (D,E) Blue points are pre-activation timepoints, dashed blue line gives expected myosin accumulation based on slope of pre-activation data points. Red points are post activation timepoints, and red dashed line shows fit of post-activation data points. Vertical grey dashed lines show the time of activation (left) and the time when the effects of activation subside (right). Fit of data points for the various segments are indicated. (F) Strain rate vs myosin rate plot of opto data points plotted over WT (See Fig 1I). Each shape corresponds to measurements from a single activated embryo from 5 embryos total. Filled and solid shapes are different regions within the same embryo. Strain and myosin rates are measured for each region before (cyan) and after (magenta) activation. (G) Strain rate plotted as a function of position along the DV axis (compare Fig1H') with the values of strain rate obtained from different experiments plotted over (red points). (H) Myosin rate plotted as a function of position along the DV axis (compare Fig1H) with the values of myosin rate obtained from different experiments plotted (red points). (G,H) Each data point corresponds to a region of an embryo similar to those shown in (A') taken from a total of 5 activated embryos, with 3-6 regions per embryo.

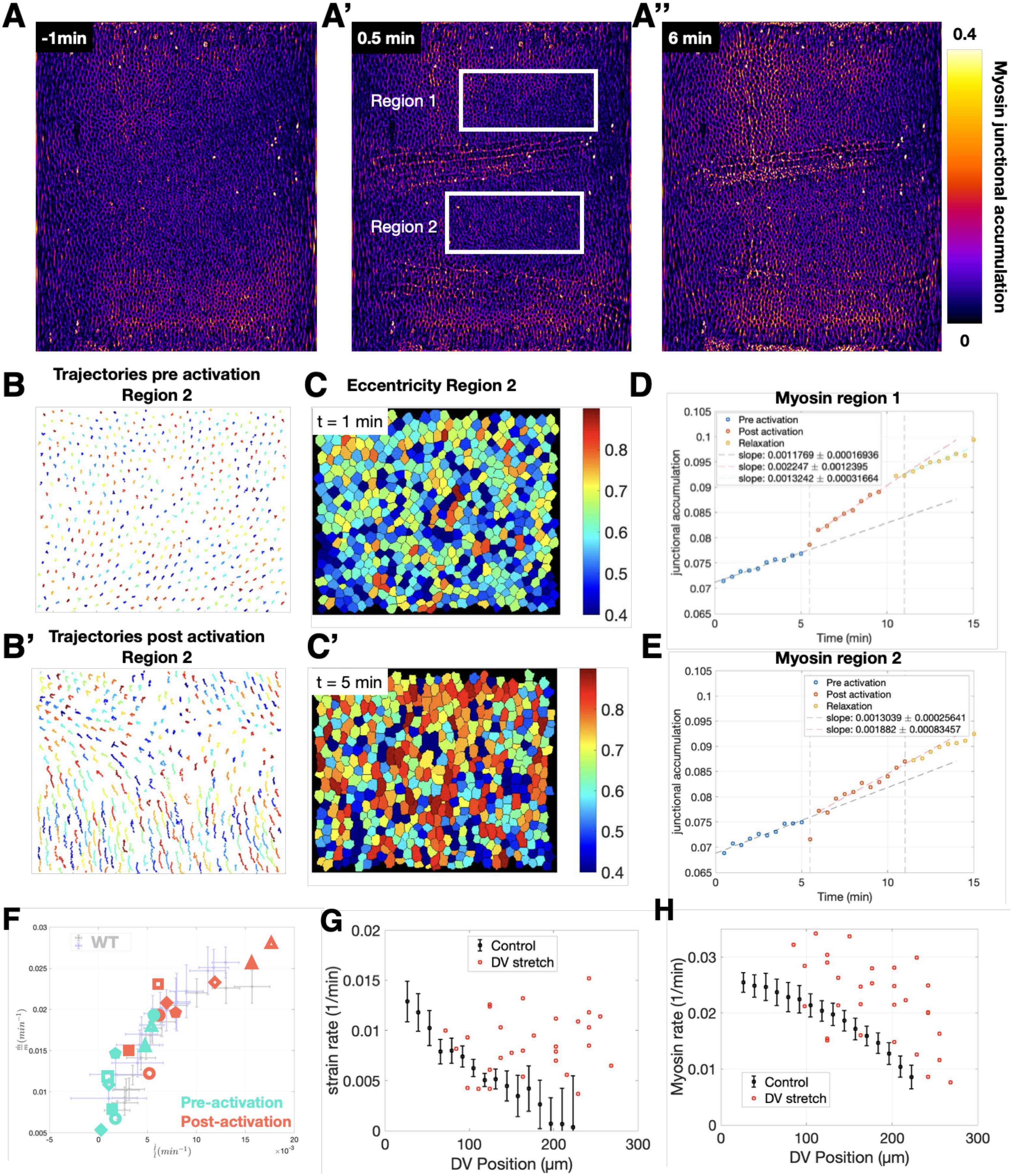

A
A'
A''
-1min
0.5 min
6 min
Region 1
Region 2
0.4
0
Myosin junctional accumulation
B
Trajectories pre activation Region 2
C
Eccentricity Region 2
t = 1 min
D
Myosin region 1
Pre activation
Post activation
Relaxation
slope: 0.0011769 ± 0.00016936
slope: 0.002247 ± 0.0012395
slope: 0.0013242 ± 0.00031664
junctional accumulation
Time (min)
B'
Trajectories post activation Region 2
C'
t = 5 min
E
Myosin region 2
slope: 0.0013039 ± 0.00025641
slope: 0.001882 ± 0.00083457
F
WT
Pre-activation
Post-activation
G
Control
DV stretch
strain rate (1/min)
DV Position (µm)
H
Myosin rate (1/min)

Figure S5: Myosin accumulation on edges parallel to AP axis during GBE

(A) First frame of a confocal time lapse image showing the germ-band of an embryo expressing a membrane marker (GAP43mCherry, sqhGFP). White dashed boxes show region tracked. Cells undergo cell intercalation resulting in a change in the region aspect ratio. Colored lines show tracks of individual cells within the region. White arrows give direction of tissue flow. (B) Three time points from the region shown in (A) towards the end of the movie showing the sqhGFP marker. Junctions parallel to the AP axis can be seen elongating and accumulation myosin. (C) Quantification of the relative myosin intensity on edges parallel to the AP axis (Blue) and their length (red).

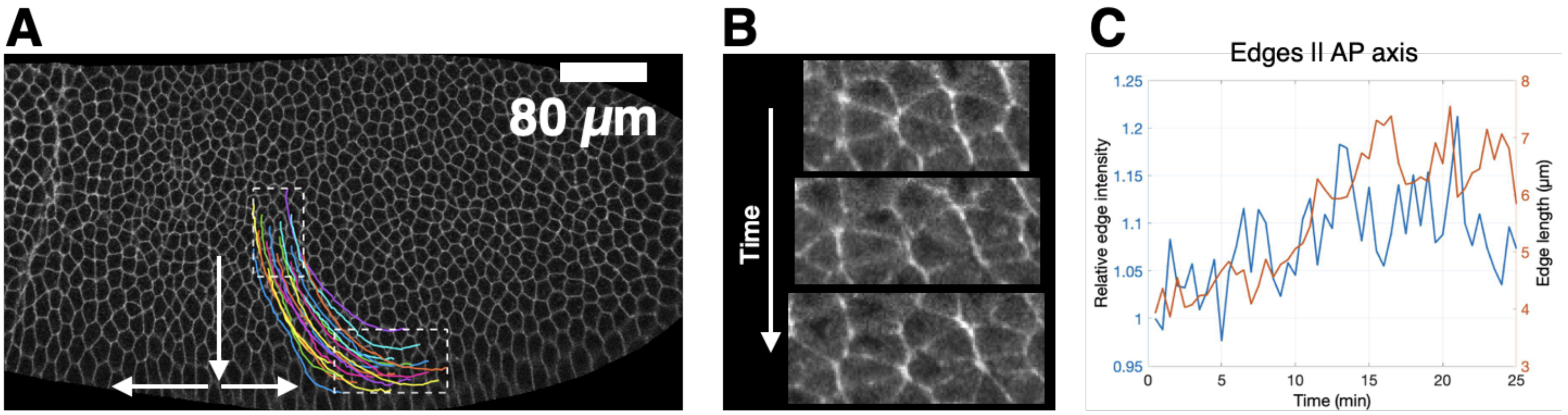
A
80 μm
B
Time
C
Edges II AP axis
Relative edge intensity
Edge length (μm)
Time (min)

Figure S6: Ventral furrow mutant analysis: Twist and Snail

(A) Junctional myosin accumulation in snail heterozygous (‘control’, A) and snail homozygous (‘Snail -/-’, A’) embryos at equivalent times. Lookup table is the same for A,A’. (B) Strain rate vs myosin rate plot with the average wild type curve (blue) and individual twist as well as snail embryos (points). Error bars are standard deviation. (C-C’’) Flow field 15 minutes post cephalic furrow formation in control (Twist heterozygous) (C), twist homozygous (C’), and snail homozygous (C’’) embryos. (D) Time course of junctional myosin accumulation in twist homozygous embryo.

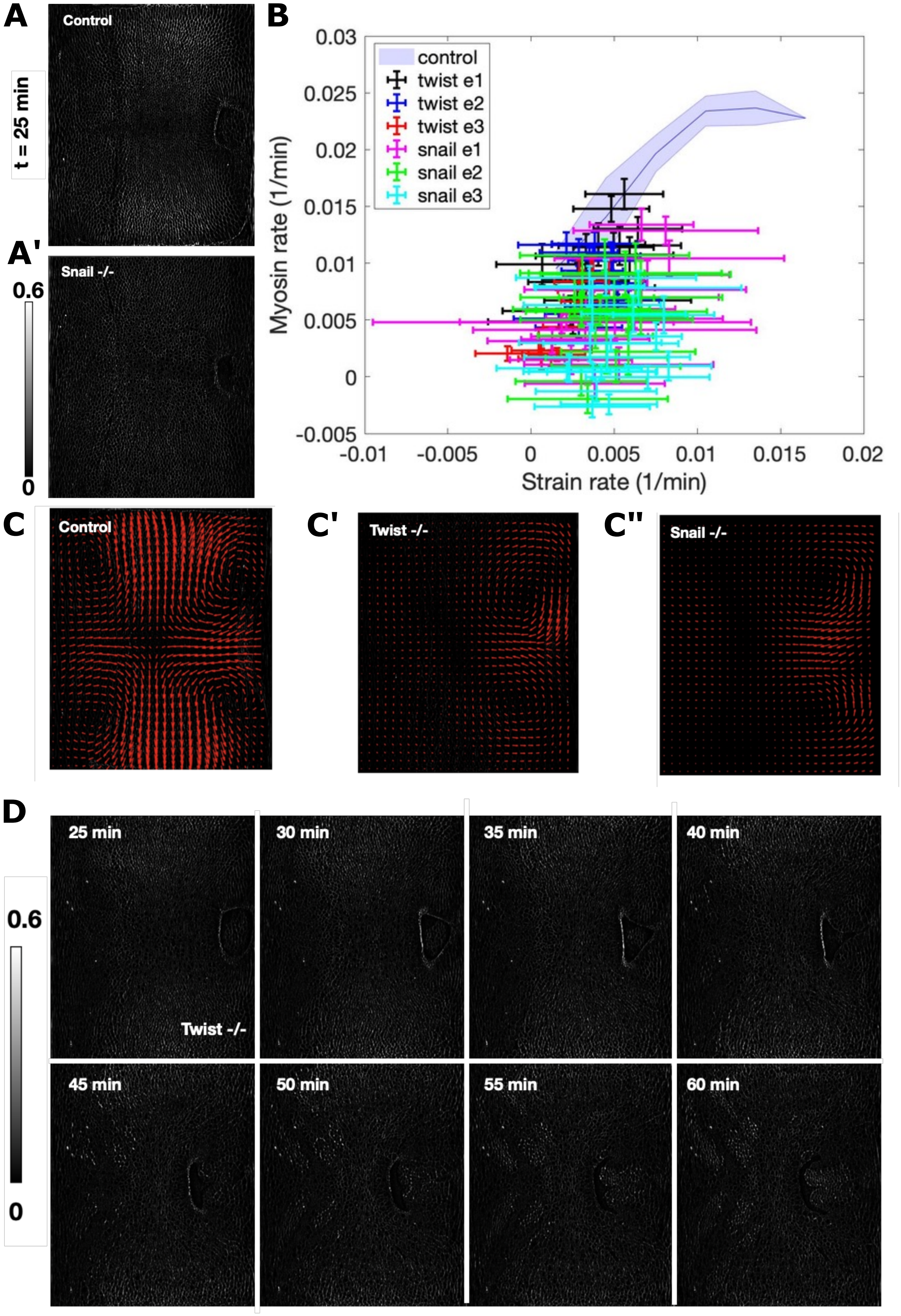
A
Control
t = 25 min
A'
Snail -/-
0.6
0
B
control
twist e1
twist e2
twist e3
snail e1
snail e2
snail e3
Myosin rate (1/min)
Strain rate (1/min)
0.03
0.025
0.02
0.015
0.01
0.005
0
-0.005
-0.01
-0.005
0
0.005
0.01
0.015
0.02
C
Control
C'
Twist -/-
C''
Snail -/-
D
25 min
30 min
35 min
40 min
Twist -/-
45 min
50 min
55 min
60 min
0.6
0

Figure S7: Concentration oscillator with mechanical feedback model and simulations
(A) Labeled illustration of extended concentration oscillator model. The elements responsible for long time dynamics are not present in the oscillator model of Dierkes et al. (2014) (8). (B) Illustration of a junction undergoing a cycle of concentration-oscillations. Each panel indicates a snapshot with time running clockwise. The green bar illustrates myosin concentration and the spring below junctional elasticity. Filled black arrows indicate movement, and curled arrows indicate net myosin binding and net unbinding. (C) Comparison of peak-aligned myosin and strain rates, for the extended (solid lines) and pure (dashed lines) concentration oscillators. Note the asymmetric response of the myosin rate in the extended model. (D) Myosin kymograph from idealized one-dimensional simulation of ventral furrow formation as described in SI section on mechanical reaction-diffusion systems.

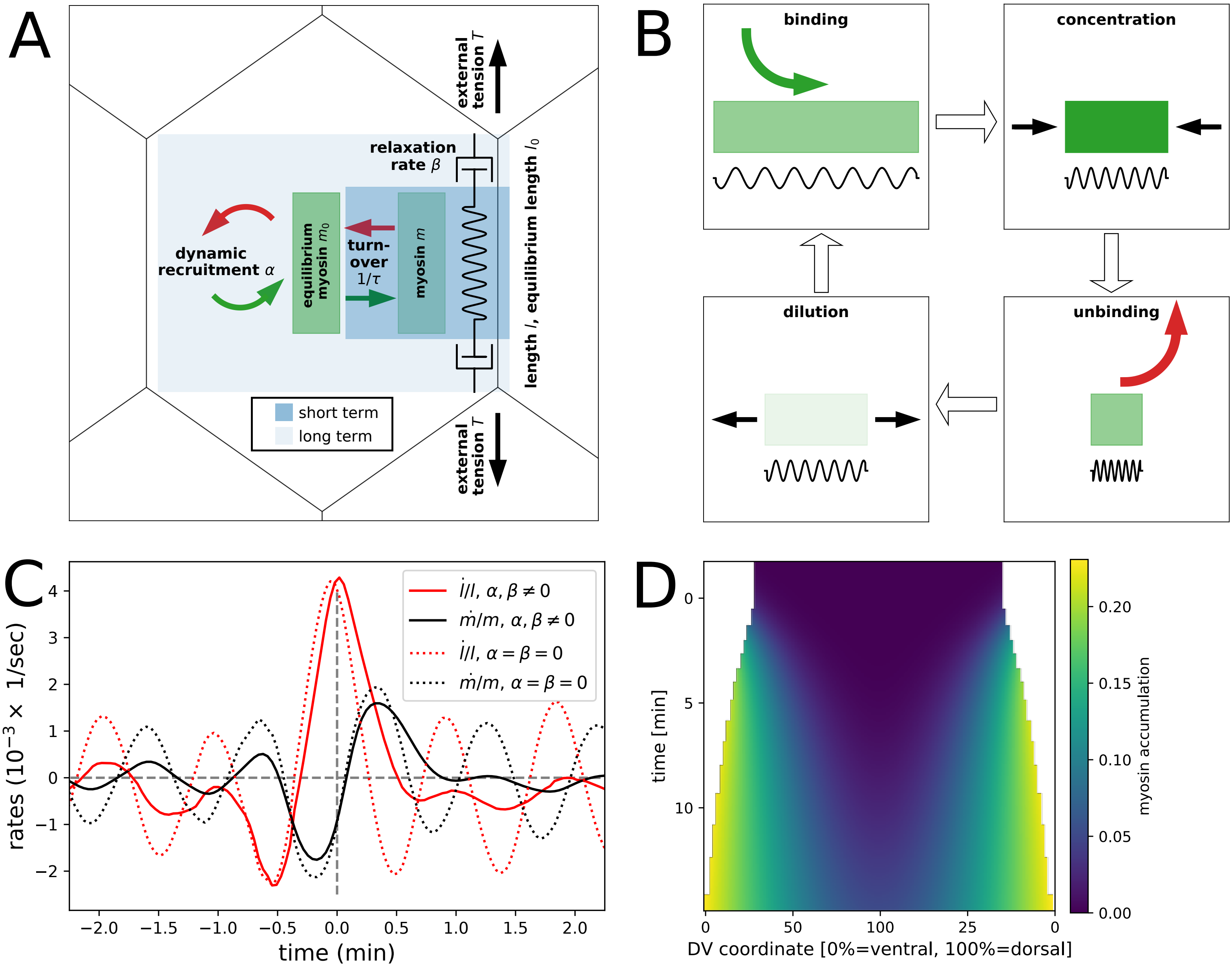
A
external tension T
relaxation rate β
length l, equilibrium length l0
dynamic recruitment α
equilibrium myosin m0
turn-over 1/τ
myosin m
short term
long term
external tension T
B
binding
concentration
dilution
unbinding
C
rates (10−3 × 1/sec)
time (min)
l̇/l, α, β ≠ 0
ṁ/m, α, β ≠ 0
l̇/l, α = β = 0
ṁ/m, α = β = 0
D
time [min]
DV coordinate [0%=ventral, 100%=dorsal]
myosin accumulation

Figure S8: Simulation results for model of tension vs strain rate dependent mechanical feedback (A) Schematic of the myosin rate detachment rate and the tension- sensitive recruitment rate as a function of the myosin level, which saturates at high myosin levels. The two stable fixed points, corresponding to low and high levels of junctional myosin are marked by black dots. The presence of two stable equilibria is called bistability. (B) Time trace of external pulling force used in simulations. The overall scale of tension is arbitrary (a choice of simulation units) and not shown. (C) Tension and the Laplacian of tension as obtained from a 1d simulation, at simulation time $t = 5$, showing different spatial distributions. (D) Mean myosin rate in stress-feedback model with external tension of varying strength. "1" corresponds to the strength used in all other simulations. (E-E') Myosin kymograph and section at $t = 40$ of the myosin distribution, simulated using strain-rate feedback (E) and stress feedback (E'). White areas indicate the "invagination"/"activation" region. (F-F') Strain & myosin rate as a function of the distance from the ventral furrow (F) and qualitative fit to the data generated using the strain - rate model Eqs. 3 and 6 (F').

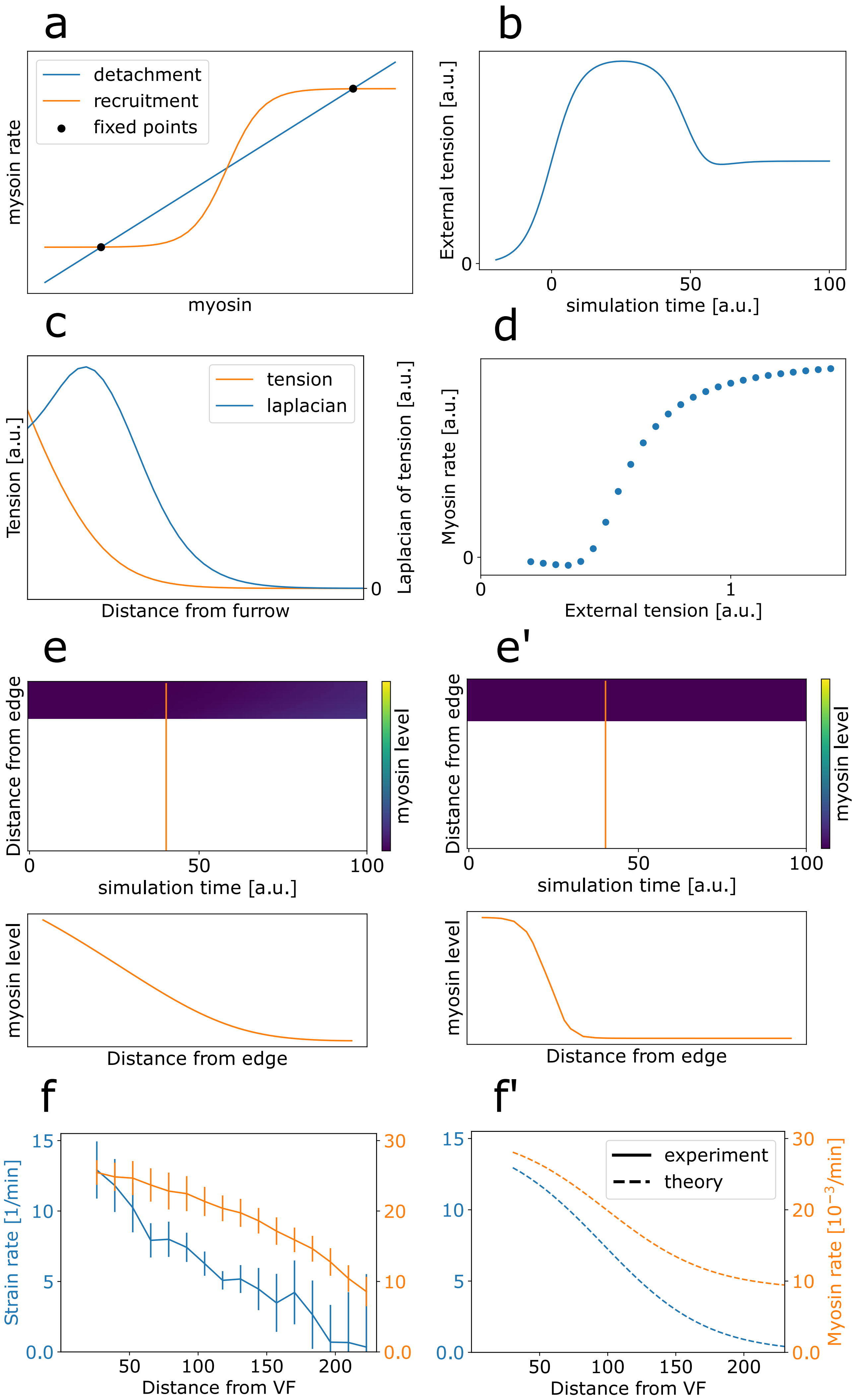

a
detachment
recruitment
fixed points
mysoin rate
myosin
b
External tension [a.u.]
simulation time [a.u.]
c
tension
laplacian
Tension [a.u.]
Laplacian of tension [a.u.]
Distance from furrow
d
Myosin rate [a.u.]
External tension [a.u.]
e
Distance from edge
myosin level
simulation time [a.u.]
myosin level
Distance from edge
e'
Distance from edge
myosin level
simulation time [a.u.]
myosin level
Distance from edge
f
Strain rate [1/min]
Distance from VF
f'
experiment
theory
Myosin rate [10^{-3}/min]
Distance from VF

Supplementary Methods

Tissue cartography at adherens junctions in light sheet microscopy movies

Pullbacks of the embryo surface are made using tissue cartography (*1*). Briefly, a pixel classification workflow in ilastik (*2*) is used to detect the embryo surface. The resulting point cloud (white dotted line, Fig S1A) is then fitted using a cylindrical bases with cross sections continuously varying in center, radius, and eccentricity as a function of the embryo's long axis. The cylinder coordinates azimuth and long axis in this way are used to generate a pullback of the fluorescence on the surface of interest. The surface can also be normally evolved in order to create pullbacks of slightly different radial distance from the embryo surface. The fitted surface is normally evolved to set a sub-apical position as the central layer (red solid line, Fig S1B), and this surface is then normally evolved by 2 pixel increments for +/- 25 steps from the central layer, for a total of 51 "onion layers' (red dotted lines show inner and outer most layers). These onion layers effectively create a 3D stack of the embryo surface moving from outside of the embryo towards the embryo center. The physical thickness of this 'stack' corresponds to 25 μm, which is small compared to the radius of curvature of the embryo. At this step, the stack is 'rotated' into a well-defined frame of orientation such that anterior is left, posterior is right, dorsal is in the center, and ventral is top and bottom. Aligning the images in this way allows for easier comparison between embryos.

We are interested in analyzing the myosin pool localizing specifically to the adherens junctions. Fitting the embryo in terms of the cylinder as outlined above has certain limitations, as the embryo does not perfectly follow the cylinder basis used in the first fitting step. Therefore, pullbacks obtained in this way have slight variations in the relative position of the apical surface of the embryo, which is somewhat exaggerated in the cartoon for clarity (Fig S1E). The top image shows a systematically varying height of the adherens junctions as a function of position within the embryo relative to the original Surface of Interest. As a result, a single onion layer passes through cells in different regions at different heights (black dashed box), and many layers would need to be analyzed to capture the junctional actomyosin network across the entire surface. This could potentially mix signals from different depths within a cell. To avoid this effect and specifically stay in the region of the adherens junction, we implemented a second fitting step. This step identifies the location of adherens junctions within the stack of onion layers. By implementing this step, the junctional myosin can be captured across the entire surface of the embryo by taking a minimal number of Z slices (Fig S1E bottom), therefore eliminating extraneous data that could affect the image analysis.

To identify the surface through the adherens junctions, we detect it as in the previous step using Ilastik, but this time applied to the stack of onion layers (white dotted line, Fig S1C). A thin plate spline fit is used to generate a smooth surface from the point cloud and a planar surface of interest defined. The surface (solid red line, Fig S1D) is normally evolved in each direction to produce 15 onion layers at 2 pixel spacing (red dashed lines represent inner and outer most layers). From these 15 layers, a maximum intensity projection of 6 layers is made which incorporates the majority of the junctional myosin signal at all regions of the embryo. This final image corresponds to a total depth of 10 pixels or 2.62 microns. In this way we obtain a thin surface of interested centered specifically around the adherens junction, showing the fluorescence signal only from within that section of the cell, across the surface of the embryo (Fig S1F)

Single junction myosin accumulation (JMA) measurements

The images described above show the fluorescence intensity of myosin specifically at the adherens junctions. In this study we use sqh::GFP and sqh::mCherry markers, which have different fluorescent proteins tagging the myosin light chain. The maternally supplied and fluorescently tagged motors localize to the cytoplasm, which functions as a large reservoir of motors that get recruited to the cell-cell interface as VF formation initiates. Although we have developed standard microscopy settings to record the data in each channel, reporting the levels of myosin on adherens junctions is best done in reference to the cytoplasmic pool. We extract the cytoplasmic pool using a top-hat transformation with a structuring element a disc of radius comparable to the cell size (Fig S1G). We then compute the ratio of the image intensity to the cytoplasmic pool (Fig S1H). This number reports the concentration of myosin at a given position in units of the cytoplasm concentration. In the cytoplasm, this would be equal to one, and greater to one on the junctions, where the density of myosin exceeds that of the cytoplasm. To obtain a simpler report, we subtract one from this result, such that the final junctional accumulation measure is zero in the cytoplasm (Fig 1C). Fig 1D&E report the average junction accumulation (i.e. the myosin density) in a local region of interest, indicated in the white box. Within this region of interest, we exclude pixel values where the junctional accumulation measure is below 0.05. Fig S1I shows a quantitative comparison of the junctional accumulation measure for two-time aligned embryos, one expressing sqh::GFP, and the other sqh::mCherry. We observe despite different markers being used, excellent agreement in the time course of the junctional accumulation measure.

For myosin rate calculation, the above protocol is applied to each timepoint. We have shown that the rate of myosin increase is linear when the VF is forming, between 10 and 20 minutes after CF formation. We therefore obtain the myosin rate by calculating the increase in junctional myosin accumulation over this time window. For all myosin measurements, with the exception of the analysis of confocal data, main text Fig 4, we use this standard measure.

Single junction quantitative analysis

Our goal for single junction analysis is to develop an automated image analysis routine that allows for extracting individual cell-cell junctions, measure their length, and report the time course of length. From this time course, we then compute the strain rate, as the change in junction length between two consecutive timepoints per time.

We start from an image showing the cell surface outline such as (Fig 1G). With ilastik, we generate a segmentation of the cells. In our images we typically find around 6000 cells during blastoderm stage. Next, we perform single cell tracking. To this end, we study the cell centroids between two consecutive frames using a point matching scheme, with the goal of mapping centroids in one time point to centroids in the subsequent one. The matching criterion is physical proximity. Cell divisions do not occur during this time, but cells can be lost from the surface due to internalization (Fig 1G). Iterating this scheme, we obtain single cell trajectories (Fig 2D). For quantitative analysis, we filter trajectories based on length of time a cell is tracked. Our movies typically cover 30 minutes of development around VF formation, typically in 60 – 120 frames. If a cell is not tracked at least 80% of the process, we discard it. In this way, we exclude around 1500 cells leaving the surface, mainly of the mesoderm, and cephalic furrow.

Next, we turn the segmentation into a polygonal tiling, where the edges of the polygon representing a cell pass through the cell surface marker (Fig 1G). In total we find approximately 18,000 edges during blastoderm stage. Using the cell tracks established as before, we turn to point

matching of the junctions within a given cell. In this way, the complexity of the problem becomes greatly reduced, instead of matching among 18,000 observations between time, we now match the number of edges of an individual cell, typically well below teens of observations. Fig 1G shows edges identified in this way using a pseudo color scheme. Fig S2 A,A' shows consecutive frames of single edge tracking performed in this way. From each of these junctions identified, we can then determine the length of the cell edge. We find the typical number of data points made in the germband region using our routine is between 250,000 – 750,000 per movie, depending on the frame rate.

Combining the panoramic overview with the tracking method outlined above, we see a majority of all shape changes at the single edge level across the embryo. Fig 1F shows striking variation in shape change across the surface, manifested in a smooth gradient of eccentricity established along the DV axis. In contrast, variability along the AP axis seems small. We therefore decided to analyze the strain rate in a regionalized way. We subdivide the DV axis into small segments covered by regions of interest that span from the cephalic furrow close to the posterior end. We compute the average edge length of all edges within each region of interest as a function of time (Fig 1G, right). This is roughly linear for the time of VF formation, so we can compute the average strain rate, i.e. the change in length of the average junction, during this time, specifically from 10 to 20 minutes after CF formation. In Fig 1H', we plot the result as a function of the DV position of the respective region of interest.

In the case of optogenetic activation experiments, large parts of the optical spectrum are blocked, as they are required for region specific activation of the opto tool. Thus, we only have the mCherry channel for observations. This channel shows the myosin marker, and segmentation of the myosin marker at the adherens junction is very challenging due to pronounced anisotropy. To obtain an automated segmentation using the same computational pipeline as described above, we study the signal on a surface slightly below the adherens junctions (4 µm), that is formed by normal evolution of the surface passing through the adherens junctions. The images on this surface show the cell surface outline, which can be readily segmented using the same ilastik process as before. Fig S2Bshows consecutive frames of single edge tracking using this strategy (compare to Fig S2A').

Optogenetic activation scheme

Here we describe our approach to generate physiological levels of tissue deformation in an epithelium of mechanically coupled cells, based on spatiotemporally controlled patterning of cell contractility. To this end, we activate an optogenetic tool capable of driving contractility inside cells, in spatially restricted domains for a transient amount of time. We refer to cells inside this domain as opto-on. Cells outside of these domains are not activated – we refer to these cells as opto-off. Upon activation of the optogenetic tool, only opto-on cells, i.e. inside of these domains, will be triggered to undergo contraction. Opto-off cells adjacent to the contracting cells will be strained by virtue of mechanical coupling, resulting in a quantitatively different strain pattern. The result of such perturbations are physiological tissue deformations very similar to those occurring naturally in the embryo, such as during ventral furrow formation (Fig 1H'). Crucially, this approach has the capability to be patterned precisely in time and space and in ways that alter the normal morphogenetic flows. In combination with the quantitative microscopy pipeline, we have developed here, we use this approach to quantitatively dissect the relation between the rate of strain on junctions and the rate of myosin accumulation.

In what follows, we will discuss 1) the opto-tool we used for activation of cell contractility and the corresponding genetic background, 2) the method we used to ensure spatial restriction of the activation pattern, and 3) the temporal pattern of activation, with reference to specific developmental processes. Except where noted, imaging was performed according to the settings described in methods section "Light sheet image acquisition".

1) To activate cell contractility, we used a two part optogenetic construct consisting of a membrane bound GFP::CIBN and a cytoplasmic Cry2::RhoGEF2 (for further details see Izquierdo, 2018). RhoGEF2, an upstream regulator of actomyosin cell contractility, is recruited to the membrane when Cry2 undergoes a conformational change in response to illumination with 488 nm light and binds CIBN. Membrane localized RhoGEF2 promotes Rho activation and subsequent actomyosin contractility. Both parts of this optogenetic construct are under UAS GAL4 control: UASp>pmGFP::CIBN and UAS>Cry2::RhoGEF2 on the second and third chromosome respectively. To drive these constructs, we used OSK>Gal4::VP16 on the third chromosome. To monitor myosin we used sqh::mCherry on the second chromosome. Specifically, we had the following genetic makeup of adult flies that we kept in the dark and used for embryo collection: w; UAS>pmGFP::CIBN/sqh::Cherry; UAS>Cry2::RhoGEF2/ osk>Gal4::VP16. We would like to note that these flies produce progeny that while kept in the dark readily undergoes embryogenesis as evidenced by hatched larvae, as described earlier (*3*). Indeed, live imaging these embryos using our light sheet microscope setup with a 561 nm laser, and keeping the opto-RhoGEF2 in the off state, we observe morphogenesis at comparable quantitative benchmarks as to control experiments without optogenetic tools (Fig S2I). From this, we conclude that the optogenetic tool does not disrupt morphogenesis in a significant way within our resolution limits.

2) Here we describe the method we used to ensure spatial restriction of the activation pattern. The activation of CRY2 CIBN interaction relies on light blue light absorption, e.g. by 488 nm. However, the *Drosophila* embryo is a spatially extended and turbid optical medium, resulting in light scattering. For faithful restricted activation in a specified region of interest, we rely on the two-photon effect, which ensures that the measured changes in myosin accumulation are not the result of stray activation. The general strategy has been explained in detail and carefully characterized in(*4*, *5*) - we recapitulate the main arguments here briefly. We use a tunable Chameleon Vision II femto-second laser set to 941 nm. This beam is split and fed into the illumination objectives, as with the other lasers, without expanding. At the energy levels output by this laser, the two-photon effect will only occur in the focal volume of the objective. Activation of the opto construct occurs by simultaneous absorption of two infrared photons. This nonlinearity effectively restricts the spatial location of activation to the well-defined domain of the objectives focal volume. In our setup, we use the excitation objective of the light sheet microscope to deliver the infrared beam in the form of a virtual sheet, generated by the same galvanometric mirror as the sheet used for regular imaging, generating a light sheet with an effective thickness of about 1 μm. However, the region of this sheet meeting the criteria for the two-photon effect is limited to the focal volume of each objective, and therefore activation effectively occurs in two regions (one for reach illumination objective) approx. 1 um wide (corresponding to the focal volume), 1 um deep, and of height determined by the amplitude of the beam scanning, generally the full height of the imaging view. Delivery of the beam to the sample is controlled via a shutter.

We used this configuration to generate the two distinct local contractility patterns outlined in the main text leading to strain parallel to the (i) DV axis, and (ii) AP axis. In both cases the

embryo is imaged for 5 minutes with a 561nm laser to obtain the "pre-activation" data (Fig S3A,E). This allows for time alignment of the experiments to control datasets, and to obtain the reference dynamics of endogenous strain and myosin rates.

For (i), we first positioned the sample at a desired position (both in terms of orientation and spatial coordinates) with respect to the imaging light sheet (Fig S3B, bright line surrounded by red box in Fig S3D,D'). We next opened the shutter controlling delivery of the femtosecond laser to the sample and moved the embryo through the sheet for five one μm steps, remaining at each step for 3 seconds, providing a total activation depth of 5-6 μm. The total activation run is completed after about 18 seconds. The shutter is then closed, concluding the activation step. The resulting myosin activation pattern 30 seconds after this procedure is shown in Fig S3D,D' from different perspectives. The myosin pattern follows an ellipse along the embryonic epithelium, a few cells wide along a roughly constant DV position both on the left and right sight of the dorsal pole.

For (ii), the sketch of the activation pattern is shown (Fig S3E,G). To activate the head region, the relative position between embryo and light sheet was modified such that the light sheet only passes through the head (Fig S3F). To ensure a uniform activation around the entire head, the embryo was imaged with 16 total positions such that the embryo was rotated and imaged every 22.5 degrees. This allows for more of the embryo to pass through the waist of the two-photon beam, which is significantly reduced compared to a single photon beam. The z step was set to 0.5 um, resulting in a total activation run of 180 seconds. Upon closing the shutter controlling the femtosecond laser, the relative position of embryo and light sheet was restored such that the sheet covers the full embryo (Fig S3G). This protocol results in nearly uniform activation in the head region with a sharp boundary delineating the non-activated trunk (Fig S3H,H'). As with the previous experiments, the activation region is clearly visible by the high intensity of cortical myosin observed 30 seconds following activation. Intensity profiles clearly show that activation is restricted to the patterned domain (Fig S3J).

3) As explain in the main text Fig 1I, we find a striking correlation between strain rate and myosin rate during the developmental period associated with ventral furrow formation. To test the causality of this relation, we timed optogenetic perturbations such that they occur between the end of cellularization, and the end of ventral furrow formation respectively. Due to technical limitations, it is challenging to more precisely time our procedure with a particular developmental time point, e.g. the first occurrence of apical constrictions in the VF, resulting in some degree of variability when exactly the optogenetic construct is activated. Our quantitative analysis strategy relies on instantaneous measurements of strain and myosin rates. Thus, absolute precise timing with respect to a common reference is not needed. All opto activation experiments are done using the spatial patterning procedure outlined above, with one single activation run (FIG S3I). Immediately after completion of this activation run, regular light sheet based imaging proceeds as for the pre-activation acquisition at 561 nm, typically covering 25 minutes of development(*6*, *7*).

Opto activation has a typical lifetime of around 5 minutes, during which myosin is recruited to junctions (*3*, *4*). From the live imaging data, we readily extract strain rates and myosin rates in cells adjacent to the activated region for the 5 minutes following activation. Together this strategy allows us to quantitatively compare these rates to those occurring in the same embryo, in the same location immediately before the activation (Fig 2B'').

High frequency single junction analysis with confocal microscopy

While light sheet microscopy allows rapid live imaging at subcellular resolution of the entire embryo, our setup has a limited temporal resolution of 15 seconds using our standard imaging settings. Confocal microscopy trades the panoramic overview for local analysis, to increase the temporal resolution by up to 10-fold. In this way, we maximize our spatiotemporal resolution, but focus on a region of interest with less cells (Fig 4A). The increased temporal resolution affords faithful computation of the correlation function between strain rate and myosin rate on the same junction, as well as probing the fast time scale response of myosin to a pull (Fig 4D,E).

For quantitative analysis of this data we proceed very similar to the light sheet microscopy data. The only difference with our analysis are the early steps. We focus the confocal microscopy in a local tissue region in a thin stack of 3μm around the adherens junctions. In this way, we do not need to perform tissue cartography preparation steps. In our setup, we image membrane and myosin channels simultaneously. Segmentation of cell outlines, and single junction tracking follows the same procedure as outlined above. For each tracked junction we can now compute the myosin line density, by averaging the junctional signal along a single pixel wide cell edge. In this way, we obtain a quantitation of both strain and myosin time course on the same junction. From this data, we directly compute the time derivative, to obtain the rates of strain and myosin respectively.

Supplementary Notes

Supplementary Note 1: Details of physical models employed in Figures 4-5

1 Concentration oscillator with mechanical feedback to describe single-edge dynamics

The single-edge dynamics observed in main text Figure 4 can be rationalized within a simple mathematical model describing the contraction and elongation of a junction under the influence of myosin contractility, viscoelasticity and external pulling forces. Myosin is concentrated and diluted as the junction shrinks respectively extends, while turnover seeks to return the myosin concentration to an equilibrium value. Over longer times, myosin can be actively and permanently recruited to the junction is response to the junction strain rate.

Above a threshold myosin concentration, the junction undergoes spontaneous short- time oscillations, which can be understood as "concentration oscillations": an initial junction contraction increases myosin density and thereby contractile forces, leading to runaway contraction eventually arrested by elasticity (in combination with non-linear effects). At this point, turnover restores the myosin level to its original value, leading over-correction and initiating the elongation phase. This is illustrated in Fig. S 7B. This mechanism has been previously proposed to explain apical area oscillations (8).

Our model allows for two additional effects, inspired by Ref. (10): At long times, the average junction length is remodeled by viscous relaxation, allowing for plastic deformation, and mechanical feedback shifts the average myosin value in response to the strain rate, adapting the junctional myosin levels to the externally imposed tension. For example, a junction under the influence of increasing external tension first deforms plastically before increased myosin levels due to mechanical feedback balance the external tension (see main text Figure 4).

This model successfully accommodates most of our observations: the main features of the autocorrelation functions, in particular their oscillatory nature and the negative correlation between strain rate and myosin rate at $\Delta t = 0$, the asymmetry in the peak-aligned autocorrelation function, as well as the correlation between strain rate and myosin recruitment seen in the lightsheet data (see main text Figure 4).

In summary, the model of junctional myosin presented in the main text can be thought of as a combination of two previously published models. At short times, it reduces to the nonlinear oscillator of Dierkes et al. (8) and at long times to the active tension network of Noll et al. (10).

1.1 Model details

Following Ref. (8), the junction is described by its length l and myosin motor concentration m. There are two further internal variable, the elastic rest length l_0 and the myosin equilibrium concentration m_0. The externally applied tension is T_{ext}. Time is denoted t. There are four dynamical equations:

$$\frac{1}{l}\frac{dl}{dt} = T_{\text{ext}}(t) - \tanh(k_3 m)/k_3 - (1 - l_0/l) \quad (1)$$

$$\frac{dm}{dt} = -\frac{m - m_0}{\tau} - \frac{m}{l}\frac{dl}{dt} \quad (2)$$

$$\frac{dl_0}{dt} = -\beta(l_0 - l) \quad (3)$$

$$\frac{dm_0}{dt} = \alpha\frac{m_0}{l}\frac{dl}{dt} \quad (4)$$

The variables and parameters of the model are illustrated in Figure S7A.

Eq. 1 describes the contraction or elongation of the junction due to three forces: external tension, myosin contractility and elasticity. Since the motion is overdamped, the strain rate $\dot{l}/l$ is proportional to the applied force. Note that Eq. (8) contains a non-linearity, in our case in the guise of a saturation of the active force as a function of motor concentration with saturation parameter k_3. This non-linearity is necessary to stabilize spontaneous oscillations and prevent junction collapse. There are multiple choices for this non-linearity, leading to similar outcomes. For example, Ref. (8) instead assumes a non-linear elastic force $-k_3(1 - l_0/l)^3$ due to strain-stiffening.

Eq. 2 describes the time evolution of myosin due to two effects: turnover of myosin with rate $1/\tau$ and matter conservation which leads to the dilution/concentration of myosin as the junction changes length. These two equations are equivalent to the model proposed in Ref. (8), with slight modifications. Note that for convenience, we have rescaled the equations so that time is measured in units of the damping constant, and myosin is measured in units of elastic tension.

Eq. 3 models viscoelastic relaxation with rate β and the junction changes shape plastically. The junctional cortex is known to behave visco-elastically due to the turnover of F-actin crosslinkers and during morphogenesis, although the exact timescale is a matter of debate.

Finally, Eq. 4, previously proposed in Ref. (10), represents mechanical feed- back with rate

α. Myosin is recruited to the junctional cortex in response to the junction strain rate. If the junction elongates as external tension overcomes myosin generated forces, additional myosin is recruited, while on a contracting junction, myosin levels are reduced. Therefore, the proposed feedback loop has the distinct property of ensuring convergence to a balanced state where external tension is compensated by myosin contractility. Following Ref. (10), we assume that mechanical feedback, an active process likely requiring the subsequent activation of several proteins, is the slowest process in the system so that $1 > \beta > \alpha$.

1.1.1 Short time behavior

At short times $t < 1/\alpha$, $1/\beta$, the above model reduces to the one of Ref. (8). We therefore briefly review the analysis presented therein. At a myosin concentration of $m_0 = 1 + 1/\tau$, the system's steady state $l = l_0$, $m = m_0$ becomes unstable to runaway contraction: junction contraction increases myosin density and thereby contractile forces, leading to runaway contraction. The system undergoes a Hopf bifurcation, with the appearance of a limit cycle corresponding to spontaneous junctional length and myosin oscillations. This limit cycle is stabilized by the non-linearity (at insufficiently high values of the non-linearity parameter, the junction collapses). The onset of oscillations at high myosin levels matches well with our observations. The model predicts that oscillations can be suppressed if the myosin lifetime τ is lowered, for example in mutant genotypes.

1.1.2 Long time behavior

On longer timescales, our model introduces two novel features, viscoelasticity and mechanical feedback (10). First, at intermediate timescales $t \sim 1/\beta$, $t < \alpha$ the system behaves according to the Maxwell model of viscoelasticity. An applied external tension $T_{ext} \neq m_0$ is initially resisted elastically before resulting in persistent flow. However, eventually, myosin recruitment adapts the equilibrium myosin concentration to the applied tension until the strain rate vanishes and $T_{ext} = m_0$. Therefore, at long times $t \sim 1/\alpha$, the junction is behaving as an effective solid, resisting applied tension. This recapitulates the active tension network behavior described in Ref. (10).

This qualitative behavior is not affected by the presence of short-time spontaneous oscillations which can be averaged over, as we have verified numerically. Interestingly, in the absence of mechanical feedback ($\alpha = 0$), the slightly asymmetric waveform of the spontaneous oscillations in combination with viscoelasticity leads to slow but persistent drift of the junction rest length l_0 (depending on the precise model parameters, the l_0 can either increase or decrease). This showcases the stabilizing role of mechanical feedback on the one hand, and on the other hand, suggests a viscoelastic dilution oscillator as a potential model for ratcheted contraction.

1.2 Simulation details

In order to create panels shown in Figure 4 of the main text, Eqs. 1-4 were solved numerically using the scientific python ordinary differential equation (ODE) solver. In all cases, the initial external tension was chosen so as to balance the initial myosin level. The non-linearity parameter was chosen to equal $k_3 = 6$, which is sufficient to stabilize the limit cycle oscillations. In all simulations, we chose the overall timescale so as to match experiment.

To calculate the temporal correlation functions, we simulated 500 junctions exposed to random tension fluctuations (Gaussian noise with short temporal correlation σ). For each simulation round, we calculated the temporal correlation functions of myosin rate and strain rate. Figure 4H shows the

average of 500 simulations. In our model, the junctional myosin level only depends on the strain rate (through Eqs. 2 and 4), and the (anti-) correlation between $\dot{l}/l$ and $\dot{m}/m$ is therefore almost maximal. The experimental data on the myosin and strain rates are subject to many other processes, random fluctuations, as well as measurement error. To model this, we added independent Gaussian noise to the simulated strain- and myosin rates, adjusting the noise amplitudes to match the magnitude of the observed magnitude of the correlation functions. The precise values of the simulation parameters c_0, τ, α, β had little influence on the final correlation functions. The parameters chosen are listed in Table 1.

The peak-aligned myosin and strain rates in Figure 4I were calculated similarly. We exposed 500 junctions to random external tension fluctuations, aligned the simulation results according to the strain rate maxima and calculated the average. In order to also model the increased tension due to optogenetic pulling forces, which induce junction elongation and myosin recruitment by feedback, we added a constant external tension exceeding the initial myosin level by 30%. The parameters used are the same as for the autocorrelation function.

To calculate the example trace shown in Figure 4, we imposed an increasing external stress $T_{\text{ext}} = T_0 + T_1 \cdot (\tanh(t) + 1)/2 + \eta$ where η represents Gaussian noise with short temporal correlation. The parameters are the same as for the autocorrelation function.

τ	β	α	k_3	σ	$c_0(t = 0)$
1	0.5	0.25	6	2	2.2

Table 1: Model parameters used in simulation. Note: overall time was rescaled after simulation to match experiment.

1.3 Comparison to concentration oscillator without mechanical feedback or viscoelasticity

To show that the asymmetric response of the myosin rate to a peak in the strain rate is indeed an effect due to the combined effects of viscoelasticity and strain-rate feedback as claimed in the main text, we simulated the peak-aligned curves shown in Figure 4I also for the case $\alpha = \beta = 0$. In this case, there is neither visco-elasticity nor strain-rate recruitment, and the equation reduce to the model considered in Ref. (8). Figure S7C shows the comparison of the two simulation results.

1.4 Comments on the choice of model

Describing complicated biological realities mathematically remains challenging. We emphasize that the model explained above is not unique and other possibilities exit (for instance regarding the choice of non-linearity in Eq. 1), with similar qualitative results. As Ref. (8) argues, spontaneous oscillation in a contractile unit with turnover is a generic phenomenon and does not depend on model details. For example, it is not relevant whether it is myosin itself which is concentrated/diluted in a dynamic junction or an upstream regulator like Rho-kinase. Similarly, the basic phenomenology of mechanical feedback - recruitment of myosin in response to strain rate and convergence to a balanced state - does not depend on depend on the particular form we propose in Eq. 4.

The model is also not meant to realistically describe the detailed biological mechanisms involved in force generation, viscous dissipation and mechanical feedback, which, in any case, are not completely understood. Further, it is clear that myosin dynamics is affected by signals other than mechanical feedback.

Finally, we initially suspected a different mechanism, whereby the oscillations are due to an effective restoring force via mechanical feedback. However, this turns out to yield incorrect temporal correlations between myosin rate and strain rate. Further, in this mechanism, biologically plausible delays between strain rate and feedback response lead to instability.

2 Prediction of tissue-scale myosin distribution from edge strain

In this section we give a detailed description of the analysis behind the strain-rate fit presented in main text Figure 5.

2.1 Theoretical model

Strain-rate based recruitment can be described by the following differential equation for the myosin concentration m on an edge of length l:

$$\frac{1}{m}\frac{dm}{dt} = \alpha \frac{1}{l}\frac{dl}{dt} \tag{5}$$

This simply says that the myosin rate is proportional to the strain rate. The feedback coefficient α is measured experimentally as shown in Fig. 3. Note that Eq. 5 is a simplified version of our full model, described in section 1, which additionally describes oscillations of the myosin rate. These oscillations are not relevant for the long-term behavior. Mathematically, Eq. 5 can be obtained from the full set of equations by averaging over one oscillation period.

Integrating Eq. 5 with respect to time from an initial time t_0 up to current time t, one finds

$$m(t) = m(t_0) \cdot \left(\frac{l(t)}{l(t_0)}\right)^{\alpha} \tag{6}$$

This means that the myosin level on an edge is proportional to its total accumulated strain $E = l(t)/l(t_0)$, raised to the power of the feedback coefficient.

We will slightly generalize Eq. 6. First, the optogenetic measurements in main text Figure 3 indicate strongly that the feedback coefficient α depends on the position y along the DV axis. From a modeling perspective, this is easy to incorporate: instead of being a single number, the feedback coefficient becomes a function $\alpha_{DV}(y)$ of the dorso-ventral position of a junction. Based on the optogenetic measurements showing a variation in the feedback coefficient by approximately one third, we use the following form:

$$\alpha_{DV}(y) = \alpha \cdot \left(1 - \frac{1}{3}\frac{y}{L}\right) \tag{7}$$

Here, L is the length of the dorso-ventral axis, and $y = 0$ corresponds to the ventral side. Second, our observations of *twist* mutants, which lack a ventral furrow and the ensuing strain, indicate that even in the absence of strain, junctional myosin levels may change, likely due to an unknown timed factor. We model this contribution by the addition of a constant c_0, independent of the junction and its position, to Eq. 6. The complete model for a single edge then reads:

$$\frac{m(t)}{m(t_0)} = \left(\frac{l(t)}{l(t_0)}\right)^{\alpha_{DV}(y)} + c_0. \tag{8}$$

The constant c_0 is fit to the data as described in the next section.

2.1.1 Coarse-grained description

Eq. 8 describes the myosin dynamics on a single edge. In Fig. 5 we are interested in the tissue-scale patterning of myosin levels, in particular along the dorso-ventral (DV) axis. To study this, we consider all successfully tracked edges in the region of interest (ROI) of a given embryo. Then, we calculate a moving average of the total strain and the myosin levels to obtain continuous functions of the DV-axis coordinate y. In particular, this averaging greatly reduces noise due to measurement error and cell-level variability. Mathematically, let $i = 1, ..., N$ denote the edges under consideration. Then, the smoothed myosin profile is given by

$$m(y,t) = \sum_i \frac{m_i(t)}{m_i(t_0)} \cdot \frac{e^{-(y-y_i(t))^2/\sigma^2}}{\sqrt{2\pi\sigma^2}}. \tag{9}$$

and similarly for the strain profile $E(y, t)$:

$$\epsilon(y,t) = \sum_i \frac{l_i(t)}{l_i(t_0)} \cdot \frac{e^{-(y-y_i(t))^2/\sigma^2}}{\sqrt{2\pi\sigma^2}}. \tag{10}$$

Note that the position y_i in Eq. 10 is the position of the edge *at time t*. σ is the smoothing kernel size, typically a few cells diameters. Since this is a linear average, the single-edge relation Eq. 8 should also hold between the spatially smoothed myosin and strain profile:

$$\frac{m(y,t)}{\overline{m}(t_0)} = \epsilon(y,t)^{\alpha_{DV}(y)} + c_0. \tag{11}$$

Here, we replaced the initial myosin distribution $m(y, t_0)$ by its spatial average $\overline{m}(t_0)$. This is justified, since the myosin profile is initially approximately constant, reduces noise, and obviates the need to track edges in the myosin data as they move along the DV axis.

2.1.2 Potential effects of finite feedback "memory"

According to Eq. 2, the myosin level on an edge depends on the total accumulated strain since the initial reference time point. However, it is biologically plausible that the cortex has a temporally finite "memory" - that is, the myosin level at time t will not "remember" strain rate experienced beyond a time $t - \Delta t$ in the past. The "memory span" Δt may (but need not) correspond to the bound or activated lifetime of a particular molecule regulating myosin concentration, for example ROCK.

The myosin profile at a given time would then reflect only the strain experience in the last Δt minutes.

A complete model for the myosin level should specify what happens to myosin after beyond the memory span time Δt. One possibility is that it might return to a background level m_0. This can for example be modeled by the following equation:

$$\frac{dm}{dt} = \alpha \frac{m_0}{l}\frac{dl}{dt} - \frac{1}{\Delta t}(m - m_0). \quad (12)$$

the solution of which is

$$m(t) = m_0 \left(1 + \alpha \int_{t_0}^{t} e^{-\frac{t-t'}{\Delta t}} \cdot \frac{1}{l}\frac{dl}{dt} dt'\right). \quad (13)$$

With our current experimental data, we are not in a position to estimate the memory range Δt. However, we note that Eq. 13 can fit the data for a range of values of Δt. This is unsurprising, since the dorso-ventral strain-rate profile is relatively constant in time during the period of observation. This means that the total strain and the strain experienced in the last Δt minutes will generally resemble one another, only differing in their magnitude. This magnitude difference can then be accommodated by fitting the feedback coefficient α.

2.2 Fit to experimental data

To evaluate this theoretical model, we need two types of data:

1. The total strain $E(y, t)$.

2. The myosin profile $m(y, t)$.

We now describe how both were obtained.

To calculate strain, cells need to be segmented and tracked. Notably, because we are interested in the total accumulated strain, and not just the strain rate, cells must be tracked over the entire observation period, requiring a good membrane marker and high frame rate. We therefore obtain the strain data from a fly line expressing CAAX- mCherry imaged with a frame rate of 15s, using the segmentation and tracking methods described in the main text. We select only vertical edges (within an angle of < 30° of the DV axis), yielding approximately 1000 edges per embryo. The continuous strain profile $E(y, t)$ is calculated from the tracking data as per Eq. 10.

To obtain the myosin profile we use embryos expressing sqh-GFP, *twist* heterozygous embryos recorded as controls for the *twist -/-* mutants shown in Fig. 5. Flies carrying the RhoGEF optogenetic construct are not perfectly suited for this purpose because even in the absence of illumination, small amounts of "leakage" cannot be excluded, which could subtly distort the myosin patterning compared to the wild type.

To extract junctional myosin from these movies, we used a somewhat simplified method compared to the main text. Segmentation is time consuming and somewhat more difficult than for the optogentic fly lines, since the sqh-GFP marker has a worse signal- to-noise ratio than the sqh-

mCherry marker in the optogentic lines. Further, to compute the smoothed myosin profile, it is not necessary to extract individual edges, and because of the averaging involved, the dorso-ventral profile is very robust to errors. Instead, we computed the intensity of the local cytosolic background I_{cyto} from the raw intensity I_{raw} and used it to extract and normalize the junctional signal: $I_{junctional} = I_{raw}/I_{cyto} - 1$. As can be confirmed by visual inspection, this isolates the junctional signal. This signal can then be averaged over the anterior-posterior axis over a region from the cephalic furrow (CF) to, but not including, the posterior midgut primordium, to obtain the dorso-ventral myosin profile $m(y, t)$.

Different lines of flies with different fluorescent markers, such as the *twist* heterozygous and the optogentic lines, will in general have different myosin background levels. Since this background level directly influences the myosin rate, the measured feedback coefficient α will differ between lines. Therefore, instead of using the value of $\alpha = 2.5 \pm 0.5$ calculated from the optogenetic lines in the main text, we fit α to get the best agreement between strain-based prediction and observed myosin levels. The dorso-ventral variation, i.e. the y-dependence of $\alpha_{DV}(y)$, is fixed by the optogenetic measurements. According to Fig. 3, the feedback coefficient is about $^1/_3$ higher on the dorsal than on the ventral pole. In between, we interpolate linearly.

The initial time point t_0 was chosen at 3-8 minutes post CF formation (i.e. to obtain $l(t_0)$ (resp. $m(t_0)$), junction lengths were averaged over this interval). This corresponds to the time just before strain is created in the ectoderm by the formation of the ventral furrow. However, the precise choice has little impact on the quality of fit. The final time point shown in Fig. 5C corresponds to the time right after the seam of the ventral furrow has closed. By then, the myosin profile has acquired its final shape, but no significant tissue flow due to germ band extension has yet occurred. The smoothing kernel size σ was chosen to equal 25μm.

2.3 Strain-rate feedback leads to mechanically mediated reaction-diffusion system

Reaction-diffusion systems are a well-established mechanism for pattern formation with clear applications to developmental biology (9). The simplest case of such a system is a diffusing morphogen, created or deposited at one position in the embryo which diffuses and establishes a gradient which is then read out by cells. Mechanical feedback, in particular if mediated by the strain rate, opens the door for mechanically mediated reaction diffusion systems: a mechanical deformation is applied at one position of the embryo, spreads through the tissue via cell-cell adherence and is read out through mechanical feedback. Since such feedback can itself recruit motor molecules and thus create forces, such systems can potentially display very rich behavior. Both mechanics and biochemistry allow for various non-linearities .

This can be illustrated by the simple case of an idealized model of the influence of the ventral furrow on myosin in the germ band, based on the equations for single- edge dynamics explained in Sect. 1. Consider a one-dimensional chain of cell edges enumerated by the index $i = 1, ..., N$. Edges will be modeled by a simplified version of Eqs. 1-4 in which oscillations are neglected and the the strain-rate feedback is linearized. Each edge has a length l_i, rest length $l_{0,i}$, strain $u_i = l_i - l_{0,i}$ and is under tension $T_i = \kappa(l_i - l_{0,i}) + m_i + T_{\text{ext}}(i)$. Edges lengthen and shorten according to tension imbalance:

$$\frac{d}{dt} l_i = \mu(T_{i+1} - 2T_i + T_{i-1}) \tag{14}$$

where μ is the viscosity. Note that the expression $T_{i+1} - 2T_i + T_{i-1}$ is a discrete approximation of the second spatial derivative or *Laplacian*, which is why we will denote it by $(\Delta T)_i$. The myosin rate is proportional to the strain rate, which is proportional to the Laplacian of tension:

$$\frac{d}{dt}m_i = \mu\alpha\frac{\overline{m}}{\overline{l}}(\Delta T)_i \tag{15}$$

Here, $\overline{m}, \overline{l}$ are the average myosin and edge length. For brevity, we define $D_m = \alpha\mu\frac{\overline{m}}{\overline{l}}$. Finally, the dynamics of the tension T_i can be worked out as well:

$$\frac{d}{dt}T_i = \mu\left(\kappa + \alpha\frac{\overline{m}}{\overline{l}}\right)(\delta T)_i - \beta(T_i - m_i) \tag{16}$$

Here, β is the rate of viscoelastic relaxation. We also define $D_T = \mu\left(\kappa + \alpha\frac{\overline{m}}{\overline{l}}\right)$. In summary,

$$\frac{dT_i}{dt} = D_T(\Delta T)_i - \beta(T_i - m_i) \tag{17}$$

$$\frac{dm_i}{dt} = D_m(\Delta T)_i \tag{18}$$

These calculations show that myosin is governed by an effective *diffusion equation*: tension applied to one end of the chain spreads, increasing myosin levels in its wake.

Eqs. 17-18 are simulated in Figure S7D for a configuration corresponding to an idealized version of the ventral furrow's pull on the germ band. The chain coordinate *i* corresponds to the position on the dorso-ventral axis, and the cable has periodic boundary conditions. The chain segment corresponding to the invaginating mesoderm is contracted, applying a constant external tension to the chain. This results in a patterned accumulation of myosin on junctions along the DV axis. In this simulation, both the strain and the myosin dynamics are computed according to the model equations, contrary to the fit presented in main text Figure 5, where the measured strain is used as input to compute the myosin pattern. This is why Figure S7D only shows qualitative agreement with the actual observed myosin pattern: the kinetics of ventral furrow formation are more complicated than captured by the one-dimensional model. In particular, the tension applied by the VF is not constant in time. Further, due to the three dimensional nature of mesoderm invagination, stress is not only directly applied to the ventral boundary of the germ band, but potentially also to regions slightly more dorsally as the internalized mesoderm presses against the germ band from below (11).

Supplementary Note 2: Strain vs. tension based mechanical feedback

Qualitative Discussion

In the present work, we study the recruitment of myosin to junctions in an epithelium subject to pulling forces on one side. To understand the mechanism underlying mechanosensitive recruitment, in particular to potentially distinguish strain-rate and stress-based myosin recruitment, it is

important to understand how mechanical signals such as the deformation rate or the stress (i.e. the tension on cell junctions) spread through the tissue. Since the external forces are applied along a single axis (e.g. the dorso-ventral axis for the case of the ventral furrow), we focus on the variation of mechanical quantities along this axis, which we call the pulling axis.

In our experiments (see Figs. 1H' and 2J), we find that the rate of deformation decreases with the distance away from the part of the tissue where external force is applied (the VF or the regions of opto-genetic activation), which indicates that there is a substantial surface friction present (if there was no surface friction, the deformation would be independent of distance). Independent measurements also indicate significant surface friction [D'Angelo 2019 finds a hydrodynamic length of ~ 28μm] (12).

To understand the mechanics of this situation, it helps to visualize the tissue as a one-dimensional chain of springs, each representing an edge, with a pulling force acting on one end of the chain. In the presence of frictional forces, a junction endpoint (i.e. a vertex) moves to the left or to the right if the pulling forces of the junctions which meet at the vertex are imbalanced. A junction shortens or elongates if its two endpoints move with different velocities. Mathematically, this means that the velocity is proportional to the spatial derivative of the stress in the tissue along the pulling axis, and that the strain rate is proportional to the second derivative of the stress (also called the Laplacian).

A simple but crucial conclusion from this is that the spatial profiles of the strain rate and the stress along the pulling axis are not the same, since the former is proportional to the second derivative of the latter. Therefore, if the myosin rate closely correlates with the strain rate in space (as shown in Figs. 1H-H' and 5B), then it cannot also correlate with stress.

In addition, there is a second line of argument which can help distinguish strain-rate and stress-based myosin recruitment. Stress-based feedback has the tendency to be unstable: stress (tension) on a junction recruits myosin, which increases stress, in turn increasing myosin levels. The stress-based recruitment must saturate at some level, given that myosin levels cannot increase to infinity, so that myosin levels plateau at a high value. Since myosin levels do not spontaneously increase, the low-myosin value must however also be stable. This situation, with two possible stable myosin levels, one high and one low, is referred to as bistability and is the generic result of a stress-based myosin recruitment mechanism. Bistable myosin dynamics have recently been studied in the context of quail primitive streak formation (13). Bistability makes two crucial predictions. First, that the (myosin) response to the (mechanical) stimulus is roughly binary: either the stimulus is sufficient to drive an edge to the stable high myosin level, or not. This is in marked contrast with our observations of a proportional response to the strain rate, even at weak stimulus strength (e.g. Fig. 5F). Second, a bistable system forms fronts and would predict a region of constant, high myosin levels near the location of the mechanical stimulus separated by a clear transition from the region of low myosin farther away. Again, we do not observe fronts but a smooth decay of the myosin rate away from the region where pulling forces are applied, in accordance with proportional strain-rate recruitment. Stable stress-based feedback is also possible, if the rate of stress-based recruitment is lower than the myosin detachment rate. However, in this case, applying a mechanical stimulus does not lead to a long-lived increase in myosin levels, which we observe.

By contrast, strain-rate feedback is both stabilizing (elongating junctions recruit myosin, counteracting the extension), but also doesn't fix the overall level of myosin, so it can create persistent patterns. The equation "myosin rate ∝ strain rate" equates rates, and therefore does not fix the underlying myosin concentration, which depends instead on the strain rate history.

Another argument which might suggest stress- rather than a strain-rate sensing, is that myosin is known to form tension-sensitive "catch bonds", binding more strongly to actin fibers if under tension. The myosin level on a junction can be increased in two ways: by decreasing the detachment rate, or by increasing the recruitment rate. The catch-bond mechanism of mechano-sensation would therefore decrease the detachment rate. However, in a separate work we measured the lifetime of myosin on junctions to be approx. 220s, independent of the junctions myosin level (14). Also, this mechanism cannot explain the ~ 2-minute delay between myosin recruitment and the application of the strain rate (Fig. 2I).

Finally, we would like to clarify that while our data and analysis supports a strong role for strain-rate based recruitment in establishing the anisotropic myosin pattern ahead of germ band extension, we do not think that the strain rate is the exclusive source of myosin. Indeed, our data supports an additional, strain-rate independent contribution. This can be seen from Fig. 1I, where we observe an increase in myosin levels of approx. 1% / min at zero strain rate. This contribution, which appears to be spatially constant during our observation period, is also taken into account in our strain-rate based prediction of the spatial myosin pattern in Fig. 5B. The contribution may well be due to stress- based feedback. Epithelial stress in the gastrulating Drosophila embryo is known to be parallel to the DV axis, and is mostly balanced (i.e. constant along the DV axis) (15). It may originate in part from the turgor pressure within the embryo, which must be balanced by epithelial stress. In a related work, we explore the possible consequences of stress-based recruitment on the myosin anisotropy orientation during germ band extension (14).

Mathematical Model

To understand the difference between tension- and strain-rate feedback we turn to a physics-based model of tension applied to one end of an epithelial sheet. While this model is idealized, the key conclusions are general. Since in our experiments, the tissue is being pulled on along a single axis, we consider a one-dimensional model for simplicity. It consists of a chain of edges $i = 1, ..., N$. The edge endpoints are denoted x_i, so that the length of each edge is $l_i = x_{i+1} - x_i$. Each edge is modeled as a spring with a "rest length" ℓ_i and a spring constant κ, so that the tension in each edge is $T_i = \kappa(l_i - \ell_i)$. ℓ_i is the rest length of the junctional actin meshwork. Edge endpoints move if there is a force imbalance, which is resisted by surface friction (with coefficient of friction γ). The influence of myosin is to work against tension to contract the actin meshwork, thereby reducing the rest length ℓ_i, with a remodeling rate β (10).

The combined equations for edge endpoint positions, tension, and the rest lengths read:

$$\gamma \frac{dx_i}{dt} = T_i - T_{i+1} \tag{1}$$

$$T_i = \kappa(l_i - \ell_i) \tag{2}$$

$$\frac{d\ell_i}{dt} = -\beta(T_i - m_i) \tag{3}$$

From Eq. 3, we can derive an equation for the strain rate *epsilon*$_i$:

$$\dot{\epsilon_i} = \frac{1}{l_i}\frac{dl_i}{dt} = \frac{1}{l_i}\left(\frac{dx_{i-1}}{dt} - \frac{dx_i}{dt}\right) = \frac{\gamma}{l_i}(T_{i-1} - 2T_i + T_{i+1}) \qquad (4)$$

$T_{i-1} - 2T_i + T_{i+1} = \Delta T_i$ is the (discrete) *Laplacian* operator. It is the discrete approximation of the second derivative with respect to the axis coordinate. We obtain the evolution equation for the tension T_i:

$$\frac{dT_i}{dt} = \frac{\kappa}{\gamma}\Delta T_i - \beta(T_i - m_i) \qquad (5)$$

The Laplacian operator makes tension spread through the tissue, analogous to heat spreading away from a source of heat. The myosin concentration m_i is measured in the same units as tension.

To complete the model, we need to describe how myosin changes over time. In the present work, we propose that myosin is recruited by the strain rate, so that the rates of myosin recruitment and edge elongations are proportional:

$$\frac{1}{m_i}\frac{dm_i}{dt} = \alpha\frac{1}{l_i}\frac{dl_i}{dt} + k_{on} \qquad (6)$$

Here, k_{on} is the potential constant contribution to the myosin rate observed in Fig. 1 I and discussed above. The alternative mechanism proposed by the referee is that myosin is recruited by tension. As explained above, such tension feedback needs to saturate. This is captured by the following equation:

$$\frac{dm_i}{dt} = -k_d(m_i - m_0) + \alpha_T \cdot \tanh\big(\lambda(T_i - T_c)\big) \qquad (7)$$

Here, m_0 is the low-myosin equilibrium level, T_c the critical tension at which tension recruitment becomes substantial, α_T the feedback strength, and the tanh-function (see Fig. S8A) ensures saturation of the feedback at high tension (λ controls the speed of saturation).

We can illustrate the points made above using simulations of this model. In these simulations, we consider a chain of $N = 100$ edges subjected to pulling forces on one end (shown in Fig. 1A), as in our optogenetic experiments and during ventral furrow formation.

In Fig. S8C we show that the spatial patterns of stress and strain rate are different, so that a correlation of the myosin rate with one precludes a high level of correlation with the other.

In Fig. S8E, we show a kymograph of the myosin accumulation in the simulation using strain-rate feedback, showing a smooth myosin gradient. In fact, the strain-rate based model can reproduce the results shown in the main text Fig. 2J, i.e. the decay of the myosin and strain rates as a function of the distance away from the boundary where strain is applied. The comparison of fit and experiment and fit is shown in Fig. S8F-F'. We varied the applied external tension parameter to fit the different runs of the experiment in which the optogenetic stimulus had different strength.

We can also see what type of myosin dynamics the model predicts in the case of stress-feedback i.e. using Eq. 7. By contrast, this leads to front formation and a sharp interface between the high and low myosin regions, Fig. S8E'. Finally, as argued above, bistable tension feedback does not lead to the proportional response of the myosin rate to the applied strain rate we observe, as shown in Fig. S8D.

Supplementary References

1. I. Heemskerk, S. J. Streichan, Tissue cartography: compressing bio-image data by dimensional reduction. *Nat Meth*. **12**, 1139–1142 (2015).
2. C. Sommer, C. Straehle, U. Köthe, F. A. Hamprecht, (IEEE), pp. 230–233.
3. E. Izquierdo, T. Quinkler, S. De Renzis, Guided morphogenesis through optogenetic activation of Rho signalling during early Drosophila embryogenesis. *Nature Communications*. **9**, 2366 (2018).
4. G. Guglielmi, J. D. Barry, W. Huber, S. De Renzis, An Optogenetic Method to Modulate Cell Contractility during Tissue Morphogenesis. *Developmental Cell*. **35**, 646–660 (2015).
5. G. Guglielmi, H. J. Falk, S. De Renzis, Optogenetic Control of Protein Function: From Intracellular Processes to Tissue Morphogenesis. *Trends in Cell Biology*. **26**, 864–874 (2016).
6. U. Krzic, S. Gunther, T. E. Saunders, S. J. Streichan, L. Hufnagel, Multiview light-sheet microscope for rapid in toto imaging. *Nature Methods*. **9**, 730–733 (2012).
7. G. de Medeiros *et al.*, Confocal multiview light-sheet microscopy. *Nature Communications*. **6**, 1–8 (2015).
8. K. Dierkes, A. Sumi, J. Solon, G. Salbreux, Spontaneous oscillations of elastic contractile materials with turnover. *Physical Review Letters*. **113**, 148102 (2014).
9. J. B. A. Green and J. Sharpe, Positional information and reaction- diffusion: two big ideas in developmental biology combine. *Development*. **142**(7): 1203-1211 (2015).
10. N. Noll, M. Mani, I. Heemskerk, S. J. Streichan, B. I. Shraiman, Active tension network model suggests an exotic mechanical state realized in epithelial tissues. *Nature Physics*. **13**, 1221– 1226 (2017).
11. M. Rauzi *et al*. Embryo-scale tissue mechanics during Drosophila gastrulation movements. *Nature Communications*. 6, 8677 (2015).
12. A. D'Angelo, K. Dierkes, C. Carolis, G. Salbreux & J. Solon, In vivo force application reveals a fast tissue softening and external friction increase during early embryogenesis. *Current Biology.* **29**, 1564-1571.e6 (2019).
13. P. Caldarelli, A. Chamolly, O. Alegria-Prévot, J. Gros, & F. Corson, Self-organized tissue mechanics underlie embryonic regulation. *bioRxiv*. 2021.10.08.463661; doi: https://doi.org/10.1101/2021.10.08.463661 (2021).
14. M. Lefebvre *et al*. Geometric control of Myosin-II orientation during axis elongation. *bioRxiv* 2022.01.12.476069 (2022) doi:10.1101/2022.01.12.476069.
15. N. Noll, S. J. Streichan, B. I. Shraiman, Variational method for Image-based inference of internal stress in epithelial tissues. *Phys. Rev. X*. **10**, 011072 (2020).
