# Supplementary material for: Patterned mechanical feedback establishes a global myosin gradient
Source: Nat Commun. 2022 Nov 17;13:7050. doi: 10.1038/s41467-022-34518-9 (PMC9672098; doi:10.1038/s41467-022-34518-9)
Supplement: Supplementary file 2 — Description of Additional Supplementary Files [file 41467_2022_34518_MOESM2_ESM.pdf]

Description of Additional Supplementary Files

File Name: Supplementary Movie 1
Description: Single cell tracking of cell edges.
